# Novel insights into cancer stem cells targeting: CAR-T therapy and epigenetic drugs as new pillars in cancer treatment

**DOI:** 10.3389/fmmed.2023.1120090

**Published:** 2023-05-18

**Authors:** Veronica Veschi, Alice Turdo, Giorgio Stassi

**Affiliations:** ^1^ Department of Surgical, Oncological and Stomatological Sciences (DICHIRONS), University of Palermo, Palermo, Italy; ^2^ Department of Health Promotion, Mother and Child Care, Internal Medicine and Medical Specialties (PROMISE), University of Palermo, Palermo, Italy

**Keywords:** CAR-T cell therapy, CAR-NK, epigenetic inhibitors, cancer stem cells, epigenetic drugs

## Abstract

Cancer stem cells (CSCs) represent the most aggressive subpopulation present in the tumor bulk retaining invasive capabilities, metastatic potential and high expression levels of drug efflux pumps responsible for therapy resistance. Cancer is still an incurable disease due to the inefficacy of standard regimens that spare this subpopulation. Selective targeting of CSCs is still an unmet need in cancer research field. Aberrant epigenetic reprogramming promotes the initiation and maintenance of CSCs, which are able to escape the immune system defense. Promising therapeutic approaches able to induce the selective inhibition of this stem-like small subset include immunotherapy alone or in combination with epigenetic compounds. These strategies are based on the specific expression of epitopes and/or epigenetic alterations present only in the CSC and not in the other cancer cells or normal cells. Thus, the combined approach utilizing CAR-T immunotherapy along with epigenetic probes may overcome the barriers of treatment ineffectiveness towards a more precision medicine approach in patients with known specific alterations of CSCs. In this perspective article we will shed new lights on the future applications of epi-immunotherapy in tumors enriched in CSCs, along with its potential side-effects, limitations and the development of therapy resistance.

## Introduction

Tumors are a miscellaneous cell composition harboring a subpopulation of stem-like cells endowed with the capability of inducing tumor progression and drug resistance. Several mechanisms have been ascribed to these phenomena including the capability to evade the immune system responses (Turdo et al., 2019). Immunological characteristics specific of cancer stem cells (CSCs) such as the expression of tumor-associated antigens (TAAs), the secretion of cytokines and/or anti-apoptotic molecules along with the upregulation of STAT3 or PI3K/AKT survival signaling pathways, are able to increase resistance to apoptosis and inhibit immune response, facilitating tumor immune escape (Codony-Servat and Rosell, 2015).

Specific aberrant epigenetic modifications in DNA methylation, histone-modifying enzymes, chromatin remodelers and long non-coding RNAs, play a critical role in initiation and maintenance of CSC compartment, thus leading to tumorigenesis (Verona et al., 2022). Treatments aimed at inhibiting the immune characteristics and the epigenetic alterations specific of CSC subset such as the immuno- and epigenetic-based therapies are thus considered as the new Frontier for the selective targeting of CSCs (D’Accardo et al., 2022; Verona et al., 2022).

Chimeric antigen receptor (CAR)-T cell therapy is among the most promising therapeutic approaches, holding an enormous potential of treating hematological disorders as well as solid tumors. This strategy is based on the *ex vivo* engineering of cancer patient T lymphocytes, in order to express a CAR selectively recognizing TAAs, and subsequent reinfusion in cancer patients. Notwithstanding the binding of engineered CAR-T cells to TAAs always culminates with the activation of cytotoxic signaling, release of granzyme, perforin and cytokines, and consequent elimination of transformed cells, several CAR structures have been developed to ameliorate the efficiency of CAR-T cell killing (Khan and Sarkar, 2022). The CAR is basically composed by an extracellular single-chain variable fragment (scFv) region, followed by a spacer and transmembrane domain and an intracellular region composed by the activation domain. Several adjustments have been made especially in the intracellular domain, which has been improved by the adjunction of i. one or multiple co-stimulatory domains (CD28, 4-1BB, ICOS or OX40), ii. transgenic sequences for pro-inflammatory cytokines release, or iii. IL-2R fragment, allowing JAK/STAT pathway activation (Sadelain et al., 2013; June et al., 2018; Khan and Sarkar, 2022). The basic CAR structure used in engineered T cells is shared by CAR-NK (Lu et al., 2021). In order to ameliorate the safety of CAR, the incorporation of a suicide gene (iC9) into the CAR construct is required to reduce the uncontrolled release of inflammatory cytokines, thus preventing the cytokine release syndrome (CRS) (Guercio et al., 2021).

CAR-T adoptive cell therapy in combination with the epigenetic reprogramming represents a powerful strategy to eradicate tumors due to the capability of overcoming one of the major challenges of cancer treatments, represented by tumor heterogeneity.

## CAR-T cell-based therapy to selectively strike cancer stem cells

CSCs downregulate the expression of the major antigens’ histocompatibility complex (MHC) class I to elude CD8^+^ T lymphocyte recognition. Thus, the CAR-T cell-based therapy by recognizing a specific TAA on cancer cells and overcoming the MHC I restriction, represents the optimal strategy to target CSCs (Sterner and Sterner, 2021). Another consideration is that standard therapies are often unable to distinguish between normal stem cells (NSCs) and CSCs, determining a phenomenon known as “on-target off-tumor toxicity.” In this context, the accuracy of CAR-T cells has been improved by engineering CAR-T cells to express the SynNotch receptor, whose binding to the tumor antigen, induces the expression of a second CAR, specific for a different tumor antigen. Double targeting CAR-T cells has indeed proved to be more precise in the killing of cancer cells while sparing normal cells (Roybal et al., 2016; Srivastava et al., 2019). This approach is particularly effective in patient’s tumors displaying a downregulation or complete loss of one specific target antigen. Likewise, tumors showing the “antigen escape” resistance patterns have been successfully targeted by tandem CAR constructs containing two scFv fragments to concomitantly bind multiple TAA (Wilkie et al., 2012).

CARs have indeed been constructed in order to recognize CSCs surface markers, usually used to identify and isolate CSCs, not shared by normal stem cells. A considerable number of anti-cancer therapies based on the use of CSC-targeting CAR-T cells are currently under pre-clinical or clinical evaluation. Pivotal examples are represented by clinical studies testing the efficacy CAR-T cells directed against the CSC surface markers EpCAM (NCT02915445; NCT03563326; NCT03013712; NCT02729493; NCT02725125), CD44v6 (NCT04427449) (Casucci et al., 2013; Porcellini et al., 2020) and c-Met (NCT01837602), involved in cancer cell metastatic potential. Other evidence demonstrated that CAR-T cells in combination with chemotherapy have been applied successfully to treat CD166 (Wang et al., 2019), CD133 (Han et al., 2021) and ROR1 (Srivastava et al., 2021) expressing tumors.

Notably, an unbiased CRISPR-Cas9 screening, on both CAR-T cells and glioblastoma stem cells, identified targetable genes whose knock out could potentiate long-term activation of CAR-T cells and glioblastoma stem cell susceptibility to CAR-T cell killing. Thus, posing CRISPR screens as a novel strategy to further enhance CAR-T cell therapeutic potency (Wang et al., 2021). Similarly, multiple CRISPR-Cas9 gene editing has been performed in CAR-T cells by deleting the *TCRα*, *TCRβ* and *PDCD1* (encoding PD-1), with the scope of decreasing the mispairing of TCR and immune-evasion. The CRISPR-Cas9 engineered CAR-T cells, further modified to express the cancer-specific synthetic transgene NY-ESO-1, displayed a durable post-infusion engraftment in the treated patients (Stadtmauer et al., 2020).

Interestingly, CAR-T cells, targeting the stemness marker GD2 in neuroblastoma, have been equipped with the suicide gene (iC9) as a safe clinical strategy to interrupt the cytotoxic activity of T cells in presence of severe adverse effects such as CRS. Moreover, GD2 CAR-T cells have been modified to express IL-7/IL-15, which significantly reduced the levels of the immune checkpoint PD-L1 on the surface of cancer cells (NCT03721068) (Perkins et al., 2015; Knudson et al., 2020). Indeed, the use of immune checkpoint inhibitors in combination with CAR-T cells therapy revealed a significant improvement in long-term remission of diffuse large B-cell lymphoma patients (NCT04381741). Of note, also CAR-NK can be considered a valid and alternative therapeutic approach (Xie et al., 2020; Christodoulou et al., 2021).

These studies open new scenarios in the field of immunotherapy due to the potential use CARs cell-based therapies, also in combination with chemotherapy, epigenetic-based or targeted therapies, as an effective strategy to disrupt tumor heterogeneity.

## Epigenetic drugs targeting cancer stem cells enhance the CAR-T therapy efficacy

Epigenetic-based therapy is an increasingly attracting field in cancer research. Epigenetic compounds such as EZH2, DNMT and HDAC inhibitors are FDA approved, while other histone-methyltransferase (HMT) or histone demethylase (HDM) inhibitors are currently in clinical trials for hematological malignancies and solid tumors. Of note, several epigenetic strategies have been reported to overcome the limits of CAR-T therapy, particularly leading to a more permissive tumor microenvironment (TME). For instance, DNMT inhibitors finely modulate the macrophages immunosuppressive activity and promote the anti-tumor activity of T cells (Stone et al., 2017), while JQ1 a BET inhibitor reduces the expression levels of PD-L1 on tumor cells and immune cells present in TME (Zhu et al., 2016). Among the major obstacles to the effectiveness of CAR-T therapy, the antigen loss and the heterogeneity of antigens may be counteracted by the combination therapy with epigenetic inhibitors (Akbari et al., 2021). Particularly, DNMT and HDAC inhibitors may result to an upregulation of TAAs and of co-stimulatory molecules such as CD40, CD80, CD86 and MHC classes I and II, on tumor cells, in particular in Acute Myeloid Leukemia (AML) cell lines (Maeda et al., 2000; Magner et al., 2000). This epigenetic-based strategy facilitates and potentiates the efficacy of CAR-T cells engineered against AML cells. Of note, EZH2 inhibitors in combination with CAR-T therapy boost the anti-tumor activity of CAR-T in Ewing sarcoma by reducing H3K27me3 mark and inducing the surface expression of GD2, a stemness marker in tumor cells (Kailayangiri et al., 2019). This epigenetic approach is aimed to increase and stabilize the expression of GD2, a tumor-sparse antigen, in Ewing sarcoma cells, in order to enable GD2-CAR T cells to target all tumor cells highly expressing GD2.

Several aberrant druggable epigenetic alterations have been linked to the initiation and maintenance of CSCs across several cancer types (Veschi et al., 2019; Veschi et al., 2020; F. et al., 2022). Compounds which induce the blockade of these specific epigenetic modifications in CSC subset including DNMT1 and DNMT3 inhibitors, HDAC inhibitors, SIRT-1 agonists and PRMT5 inhibitors are currently available in clinical or preclinical settings (F. et al., 2022). Of note, in pediatric cancers enriched in stem-like cells with high plasticity, alterations of histone modifying enzymes with non-histone targets may be specifically targetable (Veschi et al., 2017; Veschi and Thiele, 2017).

Moreover, epigenetic reprogramming has been used to modulate the differentiation state and to promote the memory phenotypes of CAR-T, to improve CAR-T infiltration and persistence, and finally as an alternative strategy to avoid their exhaustion (Akbari et al., 2021; Alvanou et al., 2023).

Overall, it is becoming increasingly clear that the epigenetic reprogramming induced by the treatment with epi-drugs may exert several effects on both CSC subpopulation and CAR-T cells through i) the impairment of self-renewal and stemness capabilities of CSCs leading to an inhibition of CSC initiation (F. et al., 2022); ii) the upregulation of TAAs specific for CSCs enabling CAR-T cells to specifically target CSCs; iii) the enhancement of the intrinsic properties of T cells by histone, DNA and miRNAs modifications promoting CAR-T cells memory phenotype and reverting their exhaustion (Alvanou et al., 2023).

**FIGURE 1:**
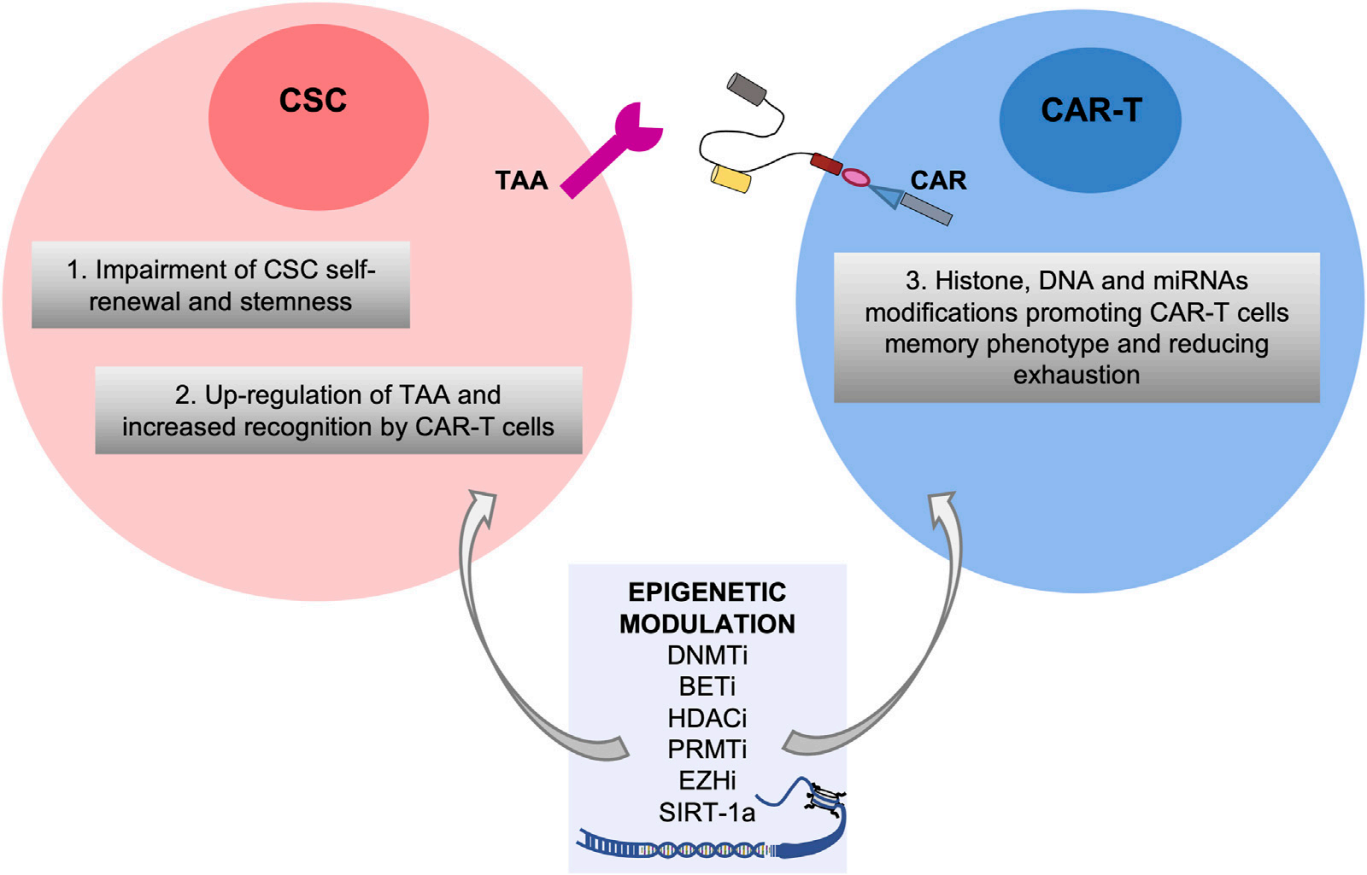
Potential effects of the combinatorial treatment of epigenetic modulators in both Cancer Stem Cell and CAR-T cell compartments. Epigenetic drugs such as inhibitors of DNMT (DNMTi), BET (BETi), HDAC (HDACi), PRMT (PRMTi) and EZH (EZHi) and SIRT-1 agonists are efficacious against aberrant epigenetic alterations of CSCs leading to impairment of self-renewal and stemness and upregulation of TAA, and on CAR-T cells promoting their memory phenotype and reducing exhaustion.

Thus, combining targeting of epigenetic alterations present on CSCs with CAR-T therapy may boost the epi-immunotherapy for cancers (Akbari et al., 2021; Xu et al., 2022) (Figure 1), although some limitations and side-effects which will be described in the next paragraphs.

## Side effects and toxicity of CAR-T cell therapy and their management in clinical practice

As a “living drug,” CAR T-cells, armed to recognize tumor antigens once infused in the blood of oncologic patients, can generate multiple side effects with diverse degree of severity. The major described effects include CRS, immune effector cell-associated neurotoxicity syndrome (ICANS) and cytopenia (Schubert et al., 2021).

CRS consists in the massive presence of inflammatory chemokines and cytokines, as IFN-γ, IL-2, TNF-α, GM-CSF, IL-8, IL-10, IL-6 and IL-2, released by activated CAR-T cells, macrophages, monocytes and dendritic cells. CRC manifests in four different grades according to symptoms severity. In order to allow an early management of CRS, temperature, blood pressure and oxygen saturation are constantly monitored. If any imbalance in these parameters is observed, a prompt CRS treatment is strictly advised, by the administration of antipyretics, corticosteroids, tocilizumab (IL-6R inhibitor) and supplemental oxygen administration (Lee et al., 2019; Neelapu, 2019).

The ICANS is observed in more than a half of patients treated with CAR-T cell therapy and can occurs independently of CRS. Patients affected by ICANS experience loss of consciousness, motor weakness and cerebral edema. According to the American Society for Transplantation and Cellular Therapy (ASTCT) grading consensus system, patients are classified in four different grades and are strictly monitored for disease evolution (Lee et al., 2019; Neelapu, 2019). Patients experiencing severe symptoms are treated with corticosteroids, mannitol, hypertonic saline and hyperventilation.

Hematological toxicity causes cytopenia in several patients treated with intravenous infusion of CAR-T cells. If persistent, autologous or allogenic stem cell transplant is recommended to cytopenic patients.

Diverse management strategies have been adopted in order to reduce mild and severe side effects sometimes requiring intensive care unit management. Safety profiles of CAR-T cell therapy have been developed primarily by lowering CAR-T cell doses.

Limitations and challenges of CAR-T cell-based therapy and epigenetic drugs targeting cancer stem cells.

Nevertheless CAR-T cell-based therapy has been successful in hematological malignancies, several obstacles have been experienced in solid tumors, besides the clinical limitations due to side effects.

The type of cancers that can be potentially targeted by both approaches, CAR-T cell-based therapy and epigenetic drugs, are ideally the same in which CAR-T alone has successfully worked: B cells malignancies, AML, chronic lymphocytic leukemia (CLL), acute lymphocytic leukemia (ALL), non-Hodgkin lymphoma (NHL) and Multiple Myeloma (MM). Of note, CAR-T cell therapy encountered several difficulties when translated into the landscape of solid tumors compared to hematologic cancers as follows: heterogeneous antigens that are difficult to be targeted, tumor physical barriers that block the CAR-T infiltration (e.g., CAFs, ECM, increased number of blood vessels), presence of several factors contributing to the immunosuppressive environment (low pH, hypoxia, immune check point blockage molecules) (Qin and Xu, 2022). In particular, the limitations of these therapies in specifically targeting the CSC compartment are based on peculiarities associated to the stem-like phenotype such as the following: 1) heterogeneous antigens potentially shared with NSCs which lead to “on target off-tumor toxicities”; 2) impaired CAR-T cells persistence and trafficking into the CSC niche in solid tumors; 3) presence of broad variety of immunosuppressive cells and molecules (Masoumi et al., 2021). On target off-tumor toxicities is determined by low levels of expression of some TAAs also in normal cells resulting in CAR-T cells targeting even the healthy cells. Recently, novel strategies to overcome the above described limitations have been proposed such as the use of CARs targeting multiple CSC antigens and the use of dual-specific CAR-T cells, optimization of the *ex vivo* CAR-T cell culture conditions, local delivery of CAR-T cells and the use of CAR-T overexpressing chemokines (Masoumi et al., 2021).

Epigenetic compounds targeting CSCs, such as HDAC inhibitors and particularly the pan-inhibitors, have been reported to induce many side effects such as diarrhea, fatigue, nausea, and anorexia. The main issue in epigenetic drug development is to identify selective compounds with significant *in vitro* cellular activity at nM concentrations without any *in vivo* toxicities. An overview about the limitations and side-effects of the epigenetic inhibitors targeting CSCs, along with the epigenetic mechanisms of resistance to therapy in CSCs, can be found in Turdo et al. (Turdo et al., 2019).

Notably, the evaluation of potential toxicities, adverse events and development of resistance induced by the combinatorial treatment based on CAR-T cells and epigenetic drugs targeting CSCs such as DNMT and HDAC inhibitors is currently ongoing in AML, ALL and NHL, although in a very limited number of clinical trials (NCT03612739, NCT04553393, NCT05797948 and NCT05370547).

Future studies will clarify these aspects and likely evaluate the efficacy of epidrugs targeting CSCs in combination with CAR-T directed against TAAs specific for CSCs.

## Conclusion

Epi-immunotherapy is a promising approach, which may overcome the challenge of specifically targeting CSC subpopulation by enhancing the immune response against the tumor cells and CSCs in particular. Notably, immunotherapy approaches based on CARs, such as CAR-T and CAR-NK, have recently revolutionized the treatment of hematological and solid tumors, enriched in CSCs.

CAR-T efficacy in combination with epidrugs is enhanced by several mechanisms which lead to an epigenetic reprogramming of both CSCs and CAR-T cells. Nevertheless, limitations and hurdles of CAR-T therapy especially in solid tumors still remain to be overcome. Several strategies to bypass these limitations, particularly the on target off-tumor toxicity, have been extensively reported. However, further studies are needed for an optimal tailoring of CAR-T cells enabling them to specifically target CSCs especially in solid tumors avoiding the well-known toxicities.

The goal of tumor eradication without recurrence may be achieved by the combination of CAR-T cells targeting TAAs specific of CSCs and epigenetic drugs specifically targeting CSCs, in association with standard radio and/or chemotherapy.

## Data Availability

The original contributions presented in the study are included in the article/supplementary material, further inquiries can be directed to the corresponding author.
